# Stretchable Porous Membranes for Barrier Tissue Models with Real-Time Measurement and Biomimetic Cyclic Strain

**DOI:** 10.3390/mi16111282

**Published:** 2025-11-13

**Authors:** Alexander P. M. Guttenplan, Joseph W. F. Robertson, Darwin R. Reyes

**Affiliations:** National Institute of Standards and Technology, Gaithersburg, MD 20899, USA

**Keywords:** interdigitated electrodes, PDMS, vacuum actuation, lithography, porous membranes, microphysiological systems

## Abstract

In recent years, the development of stretchable electronic devices with mechanical properties similar to those of human tissues has attracted increasing research interest in biomedical engineering, wearables, and other fields. These devices have demonstrated, and some other researchers have already shown, promising advancements towards applications that span from measurements of the disruption of model barrier tissues to wearable or implantable devices, soft robotics, and the development of flexible and stretchable batteries. For example, models of barrier tissues, consisting of two compartments separated by a porous membrane, have been used to measure their integrity as well as to investigate the passage of drugs, toxins, and cancer cells through these tissues. Some of these models include an elastomeric membrane which can be stretched to model processes such as breathing and gut peristalsis, while others include electrodes for real-time measurement of barrier tissue integrity. However, to date, microelectrodes have not been fabricated directly on a porous elastomeric membrane. Here, we present lithographically patterned gold electrodes on porous PDMS membranes that enable electronic sensing capabilities in addition to mechanical manipulation. These membranes are incorporated into vacuum-actuated devices which impart cyclic mechanical strain, and their suitability for electrical impedance measurements, even after 1000 stretching cycles under fluids similar to cell culture media, is demonstrated. In the future, we expect to use these electrodes to measure the disruption in model cell barriers as well as to dielectrophoretically trap cells in a region of interest for more rapid assembly of a model tissue. Other areas like wearables, robotics, and power sources will greatly benefit from the further development of this technology.

## 1. Introduction

Electronic devices and components are typically made from rigid materials such as silicon wafers and printed circuit boards. While these materials fit well into typical manufacturing workflows, their inability to stretch or bend presents challenges for the fabrication of systems which need to integrate with or mimic living organisms [1]. Polydimethylsiloxane (PDMS) is a common substrate in microfluidic and other microsystems [2], particularly at the lab research stage, as it has an elastic modulus comparable to soft tissues and its gas permeability is advantageous for devices that operate in contact with the skin for long periods [3]. Its well-attested biocompatibility with appropriate surface treatments makes it particularly attractive for biomedical research applications both in vitro and in vivo [4,5,6]. PDMS is also used in the creation of pneumatically actuated “soft robotic” systems [7], including those which incorporate electronic sensors [8].

**Figure 1:**
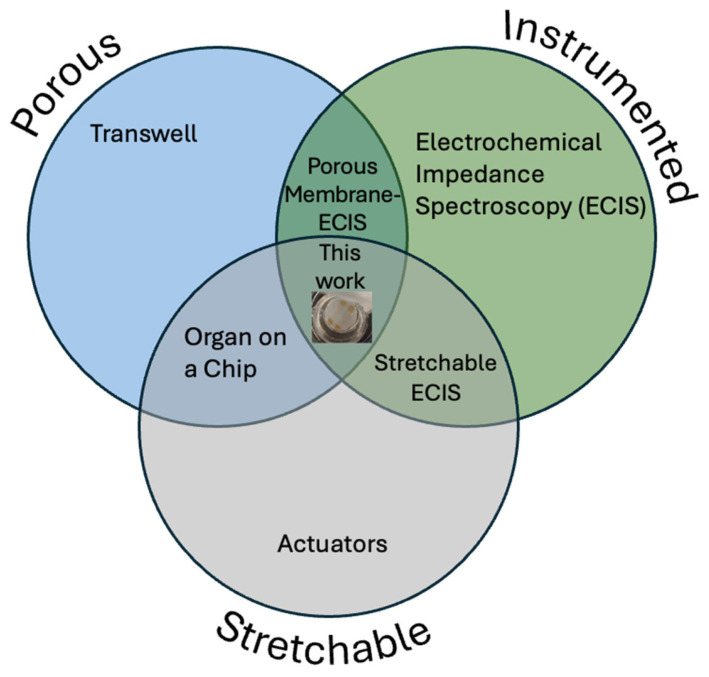
Venn diagram showing that, while porosity, actuation, and electrical measurement have previously been combined in pairs, this work is the first to combine all three.

While solid PDMS is permeable to gases and other small molecules, various methods exist to produce micropores in PDMS to increase its surface area or allow the passage of particles or cells through it. This porous PDMS has been used as a substrate for batteries [9] to enable the production of electronic devices that are fully elastic including their power supplies, and as a sponge to separate oil from water [10]. Another interesting application of porous PDMS is as a substrate for cell culture in microfabricated “organ on chip”-type devices, in particular those aiming to replicate barrier tissues [11]. The healthy functioning of complex organisms such as humans relies on a variety of biological barrier tissues acting to separate and protect the body from the outside world and to maintain homeostasis by preventing the contents of compartments within the body from mixing [12]. These barriers must also be selectively permeable to allow some exchange between the areas which they separate—for instance, the alveolar barrier in the lung must allow gas exchange between air and blood, and the gut barrier must allow nutrients to pass into the blood. Disruption in the integrity or function of these barrier tissues is linked to a range of disease states, either due to the loss of chemical homeostasis as the compartments the barrier separates move towards equilibrium with each other, or by the passage of pathogens, toxins, or immune cells into an undesired area [13,14,15,16]. In addition, researchers designing drugs must be sure that they are formulated to be able to pass through barrier tissues to reach the target organ. The use of in vitro organ- or tissue-on-chip models of barrier tissues allows researchers to quantitatively measure their integrity under different conditions. The standard in vitro barrier tissue model consists of a semi-permeable polymer membrane scaffold, such as the commercial Transwell (Certain trade names and company products are identified in order to specify adequately the experimental procedure. In no case does such identification imply recommendation or endorsement by the National Institute of Standards and Technology, nor does it imply that the products are necessarily the best for the purpose.) or Boyden chamber, on which cells are cultured to form the model tissue. The integrity of these model barrier tissues has been quantified by a range of methods, including trans-epithelial electrical resistance (TEER) as well as the transport of fluorescently labeled tracers [17]. Some model barrier tissues have also been grown on non-porous substrates incorporating electrodes, allowing the barrier function to be measured by electrical cell–substrate impedance spectroscopy (ECIS) [18,19] which is more sensitive than TEER as the electrodes are in direct contact with the cell layer [20]. This also allows the use of frequency sweeps to interrogate changes in different components of the overall cell layer impedance, such as the low-frequency response from the paracellular pathway and the high-frequency response from the transcellular pathway [21,22]. However, the lack of exposure to media on the basal side of the model barrier tissue means that this is a poor replication of the in vivo microenvironment. While incorporating electrodes directly onto a porous membrane presents a fabrication challenge [23,24], the use of such an architecture for porous membrane ECIS (PM-ECIS), which combines the biomimetic microenvironment of a porous membrane with the increased sensitivity of on-membrane electrodes, has been successfully demonstrated numerous times in the literature [20,25,26,27]. Several physiologically important barrier tissues undergo cyclic mechanical strain in vivo, such as the alveolar epithelium in the lung, which is stretched by breathing, and the gut, which is stretched by peristalsis. Other barrier tissues, such as the kidney glomerulus and the endothelium of certain blood vessels, are also under strain due to differences in fluid pressure across the barrier. This strain has been shown to affect barrier function in terms of both transport of large molecules and the migration of cells, including both cancer and immune cells. In particular, it has been suggested that inhibition of gut peristalsis adversely affects the maintenance of the gut barrier function [28]. Therefore, models have been developed which include a stretchable porous membrane as the cell culture substrate, allowing mechanical strain to be imparted to cells [29]. One common architecture for this device is a porous PDMS membrane with vacuum channels on either side of the cell culture chamber allowing it to be stretched [30,31,32,33,34,35] while other methods include external actuators [36], electromagnets [37], piezoelectric pins from Braille displays [38], and dielectric elastomers [39]. The incorporation of cyclic stretching has been shown to give a more 88 biomimetic model of the gut barrier than static models in terms of cell differentiation [40], cell organization [36], barrier function [33], and vulnerability to parasitism [41], as well as reduce the time required to form a functional model epithelium [42,43]. As the mechanical stress on barrier tissues often varies over time, whether due to cyclic stretching as in the lung or gut or due to the onset of a static pressure difference as in the kidney, it would be useful for researchers to be able to investigate the behavior of mechanically stretched model barrier tissues in real time using electrical impedance. Therefore, some groups have attempted to address the challenge of incorporating electrodes for measurement into a device in which cells are cultured on a stretchable porous membrane, such as by incorporating pairs of electrodes which are separate from the membrane [44] or using large stretchable electrodes which are not patterned [45]. However, while methods exist to pattern gold electrodes on PDMS membranes [46,47], to our knowledge the lithographic patterning of micron-scale electrodes on elastomeric membranes that are both stretchable and porous has not yet been demonstrated (Figure 1).

This novel combination would allow real-time ECIS measurements of the behavior of cells cultured on porous membranes with the mechanical stimulation of cyclic stretching. At this time, while systems for cell culture on cyclically stretched porous membranes are commercially available and widely used in research [48], they do not incorporate sensors, so cell behavior can only be monitored using microscopy or chemical analysis of the supernatant. While researchers have fabricated devices which incorporate electrodes as well as stretchable porous membranes [44,45], these do not have an electrode configuration suitable for ECIS—either the electrodes are not in the plane of the cell culture substrate, or they are very large, with a length scale of centimeters rather than microns. Similarly, while gold electrodes suitable for ECIS have been lithographically micropatterned on PDMS membranes, these membranes are not porous and, therefore, cannot be used to produce model barrier tissues under biomimetic conditions. Here, we combine transfer patterning and steam etching methods to produce porous PDMS membranes with gold electrodes lithographically patterned on their surface. These electrode-bearing membranes are then incorporated into well-plate-scale PDMS actuators, capable of stretching the membrane in a radial direction using vacuum. The electrical properties of these electrodes were measured using electrical impedance spectroscopy, showing that they are capable of measuring the difference in conductivity between phosphate-buffered saline (PBS) solutions of differing concentrations, and that the electrode traces retain their electrical continuity after a relatively large number of stretching cycles.

## 2. Materials and Methods

### 2.1. Membrane Fabrication

**Figure 2:**
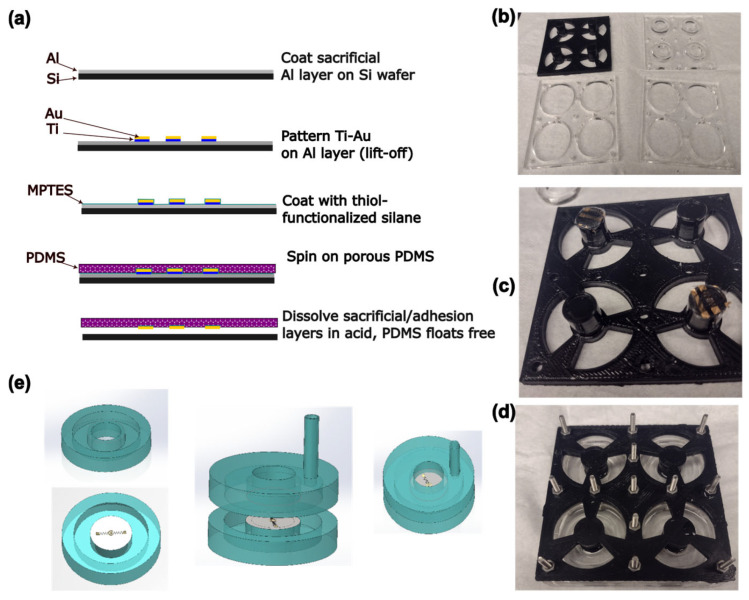
(**a**) Schematic of the process of electrode and membrane fabrication. (**b**) Photograph of the components of the mold. Diameter of large holes is 33 mm. (**c**) Photograph of membrane circles on ends of pillars before mold is assembled. (**d**) Photograph of the assembled mold. (**e**) Schematic of the process of device assembly. Device halves are fabricated separately, then bonded together.

Gold electrodes were designed using AutoCAD (version 24.3, Autodesk, San Francisco, CA, USA) and KLayout (version 0.29, https://klayout.de), consisting of a circular electrode 250 µm in diameter surrounded by a ring-shaped electrode with a 50 µm gap between the two. The electrodes were connected to 3 mm square contact pads using traces with a double meander design intended to maintain electrical continuity under strain. These electrodes were fabricated on PDMS membranes using a transfer patterning-based method previously described in the literature [46], as well as a steam etching process to generate porosity [49] through the PDMS thickness (Figure 2a). Briefly, 4″ silicon wafers (University Wafer, Boston, MA, USA) were Piranha-cleaned before a sacrificial layer of aluminum (150 nm) was deposited by electron beam evaporation (Denton Vacuum Infinity 22, Denton Vacuum, Moorestown, NJ, USA). The wafer was spin-coated on top of this sacrificial layer with LOR3A liftoff photoresist, followed by Microposit S1813 imaging photoresist (both Kayaku Microchem, Westborough, MA, USA). The resist was patterned by maskless lithography (MLA150, Heidelberg Instruments, Heidelberg, Germany) and developed using a buffered KOH-based developer (AZ 400K 1:3, AZ Microchem, Somerville, NJ, USA). Following a descum step to remove organic residues (Unaxis 790 Reactive Ion Etcher, Oerlikon, Pfäffikon, Switzerland), a ≈ 10 nm adhesion layer of titanium was deposited by electron beam evaporation, followed by a ≈ 50 nm layer of gold. The resist was then lifted off, leaving the gold electrodes patterned on the aluminum layer.

The wafer was then soaked for 2 h in a 60 mmol dm^−^^3^ solution of (3-mercapto-propyl) triethoxysilane (MPTES, TCI Chemical, Portland, OR, USA) in absolute ethanol, rinsed with ethanol, and dried with compressed nitrogen. Approximately 5 g of PDMS (Sylgard 184, Dow Chemical, Midland, MI, USA) was mixed in the recommended ratio of 10:1 base–crosslinker, poured onto the wafer without degassing, and spin-coated at 157 rad/s for 1 min. The wafer with the PDMS was then placed in a glass Petri dish and autoclaved (2340M, Tuttnauer, Hauppauge, NJ, USA) at 121 °C for 15 min to cure the PDMS and produce pores. The aluminum sacrificial layer and titanium adhesion layer were dissolved in 6% HCl overnight, allowing the membrane with the electrodes to float free of the wafer. Membranes were stored flat on Parafilm until use.

### 2.2. Device Fabrication and Assembly

PDMS actuator devices were fabricated based on the previously published FleXert design [35] (see Figure 2). The base plate and middle plates of the mold were laser cut from sheets of poly (methylmethacrylate) (PMMA) as in the original paper. However, the top plate incorporating the pillar that produces the well in the final device was instead 3D-printed by fused filament fabrication in polylactic acid (PLA) as a single piece, which was vapor-polished with chloroform [50] before use (Figure 2b). The pillar ends were recessed to prevent uncured PDMS leaking onto the membrane. Circles of membrane incorporating electrodes were cut out using a 14 mm punch and placed on the ends of two of the four pillars, with the electrode side facing away from the pillar, before the mold was assembled (Figure 2c,d). Approximately 3.7 g of PDMS (10:1 base–crosslinker, degassed) was poured into each well of the mold, and cured at 37 °C overnight, so that the membrane was monolithically integrated into the bottom halves of the devices. Holes with a diameter of 3 mm were punched into the top halves of the devices to allow vacuum tubing to be inserted for actuation. The device halves were then cleaned with isopropyl alcohol (IPA), oxygen plasma-activated (30 s, 100% power, Plasma-Preen II, Plasmatic Systems, North Brunswick, NJ, USA), pressed together using the sides of the mold as a jig, and bonded at 37 °C overnight (Figure 2e). To allow electrical connections to be made to the devices, a hypodermic needle with an outer diameter of 0.819 mm (21 G) was pushed through from the outside of the device such that it passed through both walls of the vacuum chamber and entered the well just above one of the electrical contact pads on the membrane. A tinned copper wire with a diameter of 0.254 mm (30 AWG) was threaded through the lumen of the needle until it emerged at the tip. The end of the wire was grasped with tweezers and the needle was withdrawn, leaving the wire passing through the device walls into the well. A small amount of uncured PDMS was applied to seal the vacuum chamber where the wire passed through, and cured at 37 °C overnight. Fields Metal (RotoMetals, San Leandro, CA, USA), a low-melting point cytocompatible [51] eutectic alloy, was melted and a small droplet was used to bond the wire to the electrode contact pads. A 5 mL syringe was connected to the device vacuum chamber using silicone tubing (STHT-60C, Saint-Gobain, Malvern, PA, USA).

### 2.3. Membrane Characterization

Small pieces (approx. 1 cm × 1 cm) of membrane that had not yet been mounted in a device were cut out and mounted on stubs using carbon tape, before being sputter coated with 60:40 Au/Pd and imaged using a Gemini 500 Field Emission Scanning Electron Microscope (SEM, Zeiss, Oberkochen, Germany). Images were collected of both the electrode side and the back side of the membrane, as well as of the cross-section.

### 2.4. Device Testing

A device was actuated under a microscope (Axio Observer V2, Zeiss, Oberkochen, Germany) by withdrawing the plunger of the 5 mL syringe connected to the vacuum chamber, resulting in stretching of the membrane. Images were captured with the plunger withdrawn at different amounts, and the stretching of the electrodes was measured using ImageJ (version 1.54p) [52] in order to quantify the strain in both radial and annular directions at different points on the membrane. Membrane porosity was checked by placing a device in a dish of water which reached the lower surface of the membrane, and placing a drop of water colored with food dye on top of the membrane. The food dye was observed to penetrate through the membrane into the water below. For electrical impedance spectroscopy (EIS) measurements, the contact wires were connected to an impedance analyzer (Solartron ModuLab XM, Ametek Scientific Instruments, Oak Ridge, TN, USA) using mini hook connectors. A droplet of 20 µL PBS (Gibco 1×, pH 7.4, Thermo Fisher, Waltham, MA, USA), was placed on the pair of electrodes in the center of the membrane, and the AC impedance spectrum from 1 Hz to 100 kHz was measured in order to test that the device had been assembled correctly and electrical continuity existed. This measurement was repeated three times with neat PBS and with PBS diluted 10×, 100× and 1000× with ultrapure water—the well was rinsed with ultrapure water between measurements. A volume of 1 mL was withdrawn from the 5 mL syringe, causing the membrane to stretch. The electrical conductivity was measured again in the stretched state with a droplet of PBS on the electrodes, then again after it was allowed to relax. The membrane was actuated for 10 stretching cycles using a syringe pump (PHD 2000, Harvard Apparatus, Holliston, MA, USA) and the impedance measured again with the same solutions. This was repeated after a total of 100 and 1000 stretching cycles.

## 3. Results and Discussion

### 3.1. Device and Membrane Fabrication

From the SEM images, it can be seen that pores from the combination of the steam etching effect and bubbles of air escaping from the PDMS exist on both faces of the membrane, though they are much larger on the face without the electrodes, which was directly exposed to steam during the autoclaving process and was the site of bursting bubbles of escaping air. This gradient of pore sizes has previously been reported in steam-etched porous PDMS membranes [49]. Although the cross-section images do not show a single pore extending from one face to the other, this is a function of where the membrane was cut, and the food dye tests (see Supplementary Materials) show that the porous structure is permeable. Analysis of both sides of the membrane showed that the front of the membrane (with gold) is approximately 1% to 2% porous, with an average pore size of the order of 1 μm^2^. The back (without gold) is approximately 30–50% porous with an average pore size of the order of tens of um. Both steam etching and the presence of air in the PDMS are needed for the formation of this porous structure. This is shown by the fact that if the Petri dish containing the wafer was covered during the autoclaving process, or if the PDMS was degassed in vacuum before curing, fewer or no pores formed, and those which did were much smaller (see Supplementary Materials). The mechanical properties of our porous membrane are expected to be similar to the ones reported by Jang et al. [49], where they measured the Young’s modulus of porous membranes with thickness between 40 and 120 μm. Their measurements range between 1.7 MPa and 3.2 MPa. Our membranes are expected to have similar Young’s moduli since our thickness are expected to be, based on our spin coating speed, between 50 μm and 75 μm.

### 3.2. Electrode Continuity, Actuation and Functioning

**Figure 3:**
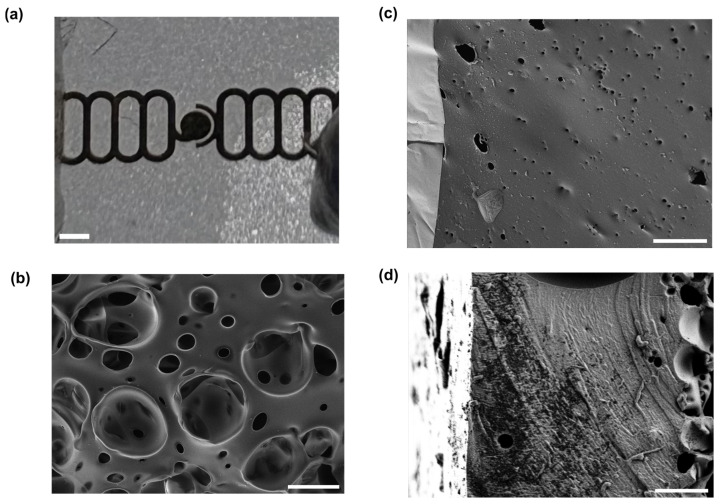
Gold electrodes on a porous PDMS membrane. (**a**) A photograph of a section of membrane with electrodes patterned on it. Scale bar 500 µm. (**b**) Scanning electron micrograph of the side of the membrane without electrodes. Scale bar 20 µm. (**c**) Scanning electron micrograph of the side of the membrane with electrodes, showing gold electrode with cracks (lighter, at left) and PDMS with pore (darker, at right). Scale bar 20 µm. (**d**) Scanning electron micrograph of a cross-section of a membrane. Scale bar 10 µm.

**Figure 4:**
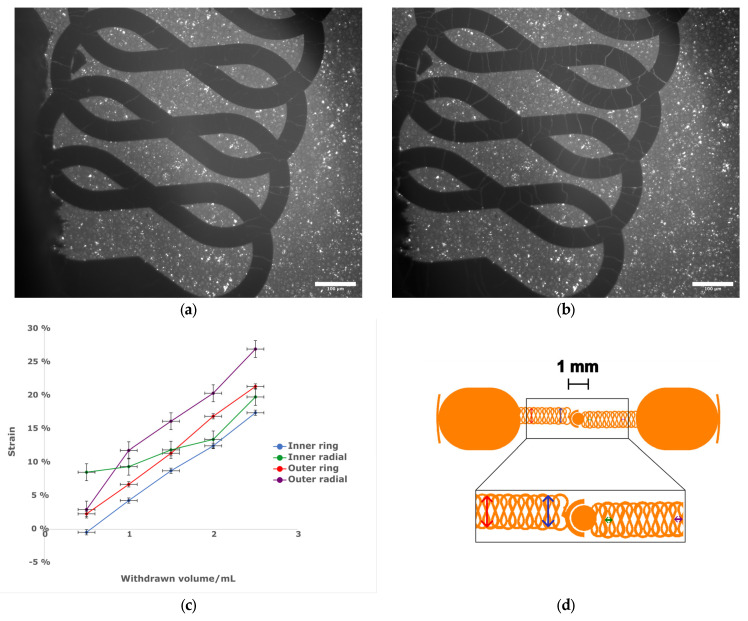
(**a**) Light micrograph of the central region of a membrane in a relaxed state without actuation. Scale bar 100 µm. (**b**) Light micrograph of the central region of the same membrane with vacuum applied by withdrawing 2.5 mL of air using a hand-held syringe. Note cracks in electrode. Scale bar 100 µm. (**c**) The relationship between measured strain and volume withdrawn. *n* = 1, error bars represent estimated uncertainty in volume measurement of syringe and distance measurement on micrograph. (**d**) (top) Schematic of electrode design, with arrows showing direction of measured strain. Scale bar 1 mm. (bottom) Expanded view of meander region to show strain measurement locations. Colors of arrows correspond to the plot lines in (**c**).

**Figure 5:**
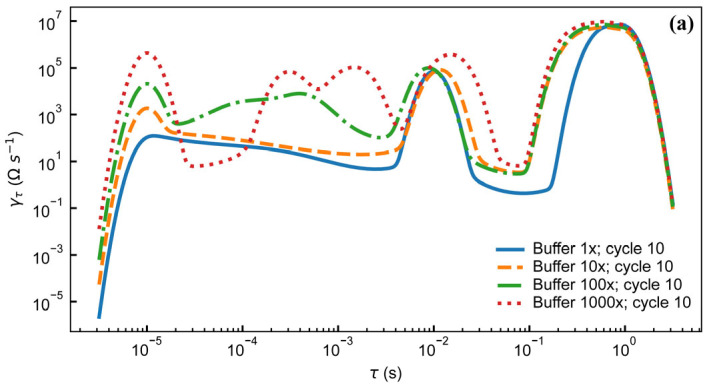

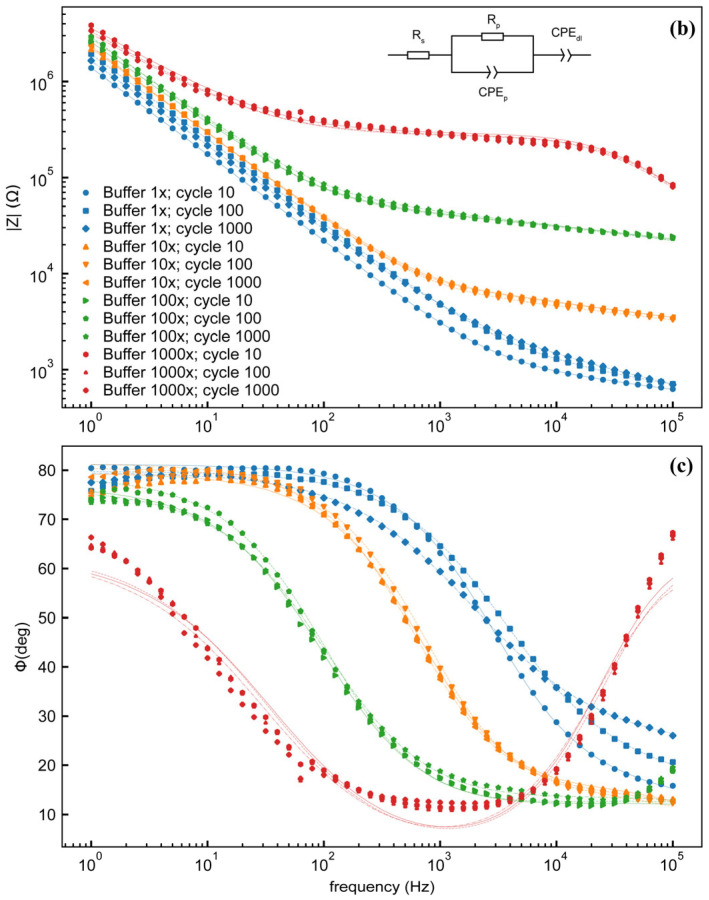
(**a**) Distribution of relaxation times analysis for electrode assembly filled with different concentrations of PBS after 10 stretching cycles. Magnitude (**b**) and phase (**c**) of electrode impedance with 4 different concentrations of PBS after 10, 100, and 1000 stretching cycles. Inset: equivalent circuit model. Legend in (**b**) applies to both (**b**,**c**). Data are from individual electrode assemblies. For uncertainty quantification, please see the table in Supplementary Table S1.

Both SEM and light microscopy images of the membrane (Figure 3) show that a continuous layer of gold has been successfully transferred to the PDMS. The width of the electrode traces is sufficiently large compared to the pores on that face of the membrane that the presence of pores does not interrupt the conducting path, and while flexing of the membrane does cause the gold to crack, these cracks do not appear to affect conductivity as the gold layers on either side of the crack overlap. Measurements of changes in electrode dimensions when vacuum is applied to the device, as seen in Figure 4, show that strain values in the range of 0–20%, comparable to those experienced by cells in soft tissues [35], can be obtained. The strain is approximately proportional to the amount of air withdrawn by the syringe pump, though some variation is seen with both location on the membrane and direction (radial vs. ring/circumferential). When the membrane is stretched under vacuum, the cracks in the gold layer open up and interrupt the conducting path (Figure 4). This is apparent both from the EIS measurement of the stretched membrane, which gives a very high impedance as seen in measurements of an open circuit (see Supplementary Materials), and from the visibly open cracks in the light microscopy image of the stretched membrane. However, when the membrane is allowed to relax and the vacuum chamber of the device is returned to atmospheric pressure, the cracks close and conductivity returns. The system impedance was measured under various electrolyte concentrations to test the electrochemical properties and stability of the electrode assembly (Figure 5). Distribution of relaxation times (DRT) analyses [53,54,55] were generated for a series of dilutions from 1× PBS to 1000× PBS (Figure 5a). At the highest electrolyte concentration, the most predominant time constant is at ∼1 s, with less prominent peaks at ∼10^−^^2^ s and ∼10^−^^5^ s, suggesting that three time constants are necessary to fit the spectra. At 10× dilution, the peaks shift to slower times indicating lower capacitance and higher resistance values in conjunction with the decreased conductivity of the solution. At 100× and 1000× dilutions, two additional time constants emerge between ∼10^−5^ s and ∼10^−3^ s, indicating additional complexity in the system when the electrochemical double-layer is large. To extract trends in these data, we use a three-time constant equivalent circuit model (Figure 5b) inset in which the solution resistance (*R_s_*) is in series with a surface transport layer modeled with a parallel resistor (*R_p_*) and constant phase element (*CPE_p_*), where the impedance of the CPE is *Z* = −1/(*Q*(*iω*)*^α^*. *Q* has units of farad (F) and *α* is a dimensionless number between 0 and 1. This is in series with a second CPE, *CPE_dl_*, which represents the electrochemical double layer. CPEs are used for two reasons in the model. The geometry of the system dictates that a potential gradient will develop across the interconnect in contact with the solution, and as such the element given by *R_p_CPE_p_* will describe ion transport perpendicular to the surface between the two electrode pads and their solution-exposed leads. The double-layer element *CPE_dl_* allows for a rough surface to have a distributed behavior. As the EIS spectra were collected under conditions where there was no redox reaction, the double layer does not have a charge transport resistance. Ignoring the additional time constants in the 100× and 1000× case, this model fits the impedance data well, with only a relatively poor phase response at the highest dilution (Figure 5b,c). The aggregated parameters for each condition can be found in Table S1. The most critical result of these experiments is the “self-healing” nature of the electrodes. When the impedance is measured while a stretching force is applied to the surface, the resulting spectrum is an open circuit (See Supplementary Materials). Impedance spectra were collected after 10, 100, and 1000 stretching cycles to investigate degradation of the electrode. For each condition, there are few systematic changes in the observed parameters outside the ±1 standard deviation error. With the highest ionic strength, there does appear to be small changes in both parallel parameters (*R_p_*, *CPE_p_*) and the double-layer parameter (*CPE_dl_*). Taken together, these shifts suggest that the electrodes change slowly over time, only observed at high ionic strength. However, the lack of systematic change in the observed parameters suggests that electrode failure will be stochastic rather than deterministic but with no failure observed in 1000 cycles, we do not anticipate electrode failure to limit their effectiveness for measurement of cell behavior in model barrier tissues that incorporate cyclic stretching. In addition, we estimated the number of peristaltic waves over a period of 24 h in the large intestine where colon cells reside. Colon cells, including Caco-2 cells and others, are the type of cells commonly used to model the gut in drug discovery and toxicity experiments, both in conventional and microphysiological systems [56]. It has been observed that contractions of this type have a frequency of approximately three contractions per minute, and these contraction cycles can last up to 30 min. Taking into account that the occurrence of these events is reported to be between 2 and 4 times every 24 h, we calculated that the maximum number of contractions would be approximately 360 per day [57,58]. Thus, testing our system for up to 1000 stretching cycles provides us with three times more stretches than would be needed to obtain results from acute toxicity experiments, which are run for up to 24 h [59]. This ensures a safe margin of error regarding the life of the device when used with gut cells and mechanical stimuli for assessing the efficacy and toxicity of new drugs.

## 4. Conclusions

Here, we have demonstrated the successful lithographic patterning of gold electrodes on a porous elastomeric membrane, which was incorporated into a well-plate-scale cell culture device. This devices could be actuated automatically using commonplace syringe pumps, and produce a physiologically relevant strain on the cell culture substrate. While the electrodes, despite their meander structure, do not retain their conductivity on stretching, the conductivity returns once the strain is removed, and is robust over large numbers of stretching/relaxation cycles. In the future, the ability of the device to record changes over time in the barrier function of a cell monolayer cultured on the membrane could be tested. In particular, the effect of cyclic stretching on the rate of formation of model barrier tissues, and the response to chemical stimuli of model barrier tissues grown in the presence and absence of stretching, could be studied. As the culture of cells on porous PDMS membranes under cyclic stretching has become routine in the field of organ-on-chip research, and cell monolayers have also been successfully cultured on non-porous PDMS membranes on which gold electrodes are fabricated [47], the combination of these features should not pose major problems for the biocompatibility of the system. Automation of actuation and measurement would both allow stretching to be paused to ensure that all measurements are taken with the electrodes in a relaxed state, and to allow larger amounts of data to be collected over time. Therefore, this device would allow more detailed and accurate measurements of the behavior of model barrier tissues under cyclic strain than are possible with existing devices that do not incorporate on-membrane electrodes. In addition, the combination of a porous PDMS membrane with electrodes is suitable for other applications, including, but not limited to, wearable electronics and batteries.

## Data Availability

The original contributions presented in this study are included in the article/Supplementary Materials. Further inquiries can be directed to the corresponding author.

## Supplementary Materials

The following supporting information can be downloaded at: https://www.mdpi.com/article/10.3390/mi16111282/s1, Figure S1: Membrane percolation test with food dye; Figure S2: SEM image of non-porous membrane produced with degassed PDMS; Figure S3: EIS of stretched membrane showing open circuit; Table S1: Electrochemical Parameters.
