# Supplementary material for: Stretchable Porous Membranes for Barrier Tissue Models with Real-Time Measurement and Biomimetic Cyclic Strain
Source: Micromachines (Basel). 2025 Nov 13;16(11):1282. doi: 10.3390/mi16111282 (PMC12654284; doi:10.3390/mi16111282)
Supplement: Supplementary file 1 [file micromachines-16-01282-s001.zip › micromachines-3850177-supplementary.pdf]

Supplementary Materials

Stretchable Porous Membranes for Barrier Tissue Models with Real-Time Measurement and Biomimetic Cyclic Strain

Alexander P. M. Guttenplan †, Joseph W. F. Robertson and Darwin R. Reyes *
National Institute of Standards and Technology, Gaithersburg, MD 20899, USA;
agutten@gmail.com (A.P.M.G.); joseph.robertson@nist.gov (J.W.F.R.)
* Correspondence: darwin.reyes@nist.gov
† Current address: United States Mint at Philadelphia, Philadelphia, PA 19106, USA.

a
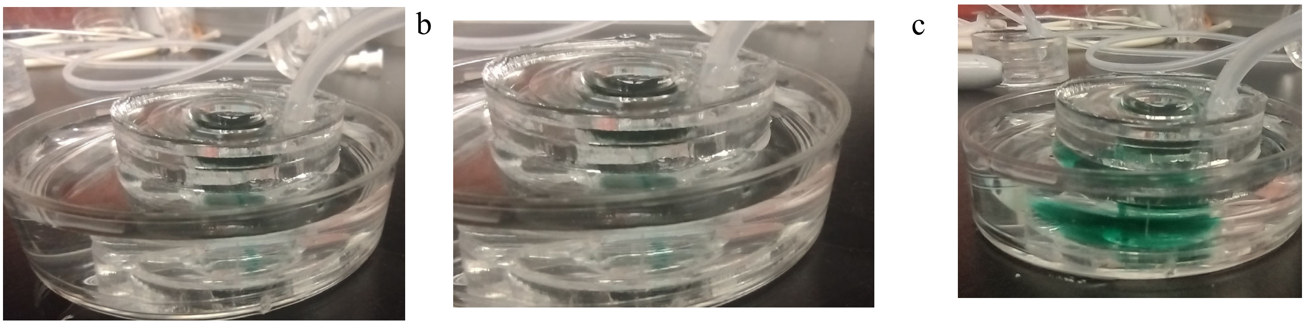

Figure S1. Food dye percolating through the porous PDMS membrane into the lower part of the well-plate device. Photographs taken (a) 5 minutes, (b) 10 minutes and (c) 30 minutes after food dye was added to upper part of device.

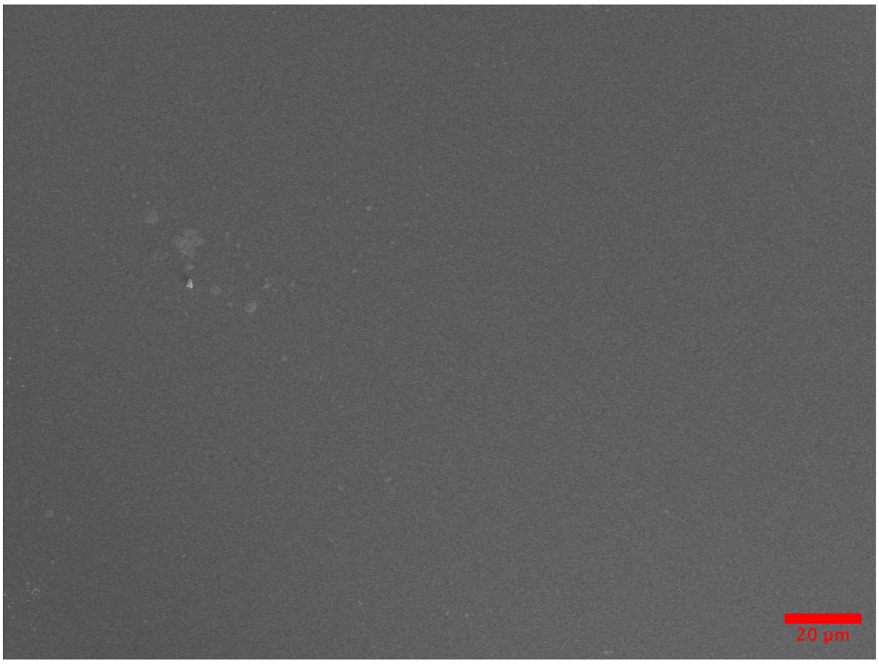

Figure S2. SEM image of the back side (without electrodes) of a PDMS membrane produced using degassed PDMS. Note the lack of pores. Scale bar 20 μm.

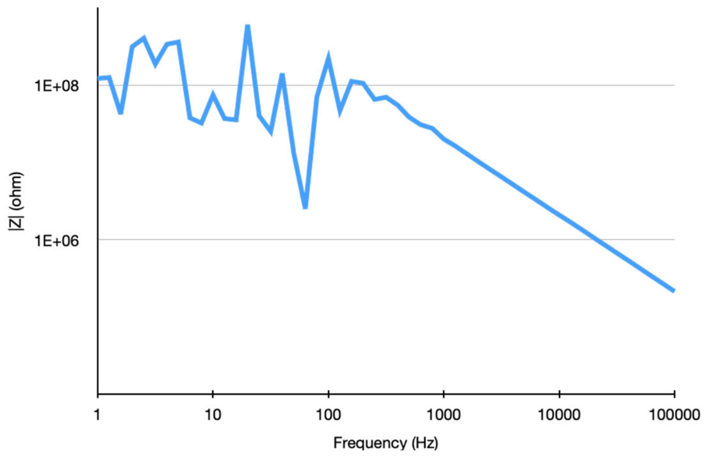

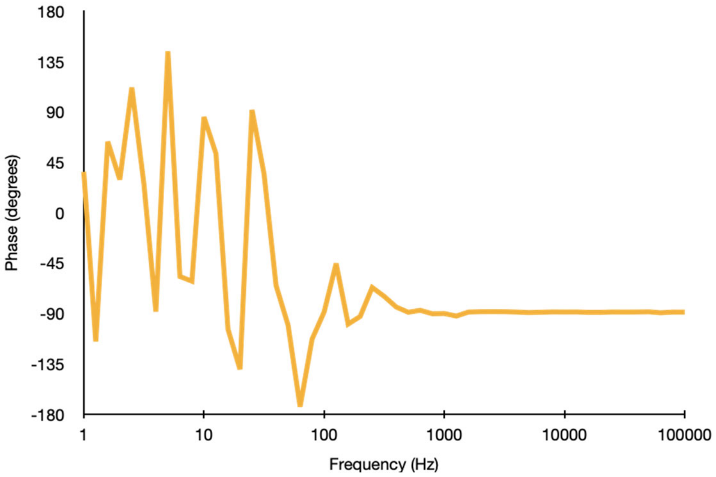

Figure S3. Plots of magnitude (top) and phase (bottom) of impedance of electrode when device is stretched, showing open circuit response.

Table S1. Electrochemical Parameters with Buffer and Cycle Variations. *Notation refers to the dilution level. †All values reported as mean ± standard deviation of three independent membranes.

Buffer *	Cycles	R_1 (Ω) †	R_2 (Ω) †	Q_2 (F) †	$α_2$ †	Q_s (F) †	$α_3$ †
1x	10	$(2.05 \pm 1.3) \times 10^{2}$	$(1.39 \pm 0.592) \times 10^{3}$	$(2.45 \pm 1.78) \times 10^{-5}$	0.34 ± 0.101	$(1.42 \pm 0.145) \times 10^{-7}$	0.889 ± 0.0115
1x	100	$(2.01 \pm 0.732) \times 10^{2}$	$(3.4 \pm 3.73) \times 10^{3}$	$(1.19 \pm 0.102) \times 10^{-5}$	0.365 ± 0.00784	$(1.15 \pm 0.251) \times 10^{-7}$	0.883 ± 0.00568
1x	1000	$(1.81 \pm 1.53) \times 10^{2}$	$(5.08 \pm 1.58) \times 10^{3}$	$(1.08 \pm 1.24) \times 10^{-5}$	0.421 ± 0.14	$(1.25 \pm 0.074) \times 10^{-7}$	0.861 ± 0.0322
10x	10	$(2.35 \pm 0.108) \times 10^{3}$	$(4.03 \pm 0.288) \times 10^{3}$	$(9 \pm 3.02) \times 10^{-7}$	0.496 ± 0.03	$(9.64 \pm 0.612) \times 10^{-8}$	0.884 ± 0.00273
10x	100	$(2.28 \pm 0.0327) \times 10^{3}$	$(3.93 \pm 0.431) \times 10^{3}$	$(8.88 \pm 0.984) \times 10^{-7}$	0.492 ± 0.0161	$(8.13 \pm 0.35) \times 10^{-8}$	0.895 ± 0.000724
10x	1000	$(2.27 \pm 0.105) \times 10^{3}$	$(5.38 \pm 0.465) \times 10^{3}$	$(8.38 \pm 5.82) \times 10^{-7}$	0.5 ± 0.0527	$(8.24 \pm 0.12) \times 10^{-8}$	0.885 ± 0.017
100x	10		$(4.62 \pm 0.294) \times 10^{4}$	$(7.23 \pm 0.941) \times 10^{-7}$	0.323 ± 0.00723	$(8.13 \pm 0.284) \times 10^{-8}$	0.86 ± 0.00657
100x	100		$(4.09 \pm 0.268) \times 10^{4}$	$(5.98 \pm 1.77) \times 10^{-7}$	0.343 ± 0.0148	$(6.85 \pm 0.216) \times 10^{-8}$	0.869 ± 0.00877
100x	1000		$(5.45 \pm 0.486) \times 10^{4}$	$(7.96 \pm 0.662) \times 10^{-7}$	0.327 ± 0.00533	$(7.19 \pm 0.327) \times 10^{-8}$	0.865 ± 0.0158
1000x	10		$(2.69 \pm 0.0613) \times 10^{5}$	$(7.18 \pm 1.22) \times 10^{-11}$	0.901 ± 0.0133	$(8.14 \pm 0.189) \times 10^{-8}$	0.705 ± 0.0107
1000x	100		$(2.43 \pm 0.187) \times 10^{5}$	$(7.74 \pm 2.61) \times 10^{-11}$	0.898 ± 0.029	$(8.51 \pm 0.698) \times 10^{-8}$	0.739 ± 0.0356
1000x	1000		$(2.66 \pm 0.107) \times 10^{5}$	$(1.08 \pm 0.413) \times 10^{-10}$	0.878 ± 0.0366	$(8.33 \pm 1.47) \times 10^{-8}$	0.716 ± 0.0309
